# Enhanced cellular uptake of size-separated lipophilic silicon nanoparticles

**DOI:** 10.1038/srep43731

**Published:** 2017-03-08

**Authors:** Aubrey E. Kusi-Appiah, Melanie L. Mastronardi, Chenxi Qian, Kenneth K. Chen, Lida Ghazanfari, Plengchart Prommapan, Christian Kübel, Geoffrey A. Ozin, Steven Lenhert

**Affiliations:** 1 Department of Biological Science, Florida State University, Tallahassee, Florida, USA; 2 Department of Chemistry, University of Toronto, Toronto, Canada; 3 Department of Physics, Florida State University, Tallahassee, Florida, USA; 4 Institute of Nanotechnology and Karlsruhe Nano Micro Facility, Karlsruhe Institute of Technology, Karlsruhe, Germany.

## Abstract

Specific size, shape and surface chemistry influence the biological activity of nanoparticles. In the case of lipophilic nanoparticles, which are widely used in consumer products, there is evidence that particle size and formulation influences skin permeability and that lipophilic particles smaller than 6 nm can embed in lipid bilayers. Since most nanoparticle synthetic procedures result in mixtures of different particles, post-synthetic purification promises to provide insights into nanostructure-function relationships. Here we used size-selective precipitation to separate lipophilic allyl-benzyl-capped silicon nanoparticles into monodisperse fractions within the range of 1 nm to 5 nm. We measured liposomal encapsulation and cellular uptake of the monodisperse particles and found them to have generally low cytotoxicities in Hela cells. However, specific fractions showed reproducibly higher cytotoxicity than other fractions as well as the unseparated ensemble. Measurements indicate that the cytotoxicity mechanism involves oxidative stress and the differential cytotoxicity is due to enhanced cellular uptake by specific fractions. The results indicate that specific particles, with enhanced suitability for incorporation into lipophilic regions of liposomes and subsequent *in vitro* delivery to cells, are enriched in certain fractions.

There are an enormous number of possible nanoparticles resulting from combinations of composition, size, shape, surface and defects, and cell specific biocompatibility has been found to depend on these nanoparticle parameters^1^,^2^,^3^,^4^,^5^. Most synthetic methods for producing nanoparticles, however, result in products that are hydrophobic^6^,^7^ in addition to having considerable polydispersity^2^,^8^. The polydispersity results in an inhomogeneous broadening of the spectral features, thereby producing averaging effects in biomedical imaging applications and cytotoxicity studies^2^,^8^. Monodisperse samples are hence necessary to narrow the optical, electrical, and biological properties. Various methods exist for the post-synthetic purification and size-separation of nanoparticles, including size-selective precipitation^9^,^10^, filtration, size exclusion chromatography^11^,^12^, and very recently, density gradient ultracentrifugation, all of which allow for the isolation of relatively monodisperse fractions of ligand-passivated silicon nanocrystals (ncSi)^2^,^13^,^14^.

While the chemical and physical properties show variation with nanoparticle size, there is also growing evidence that nanoparticle internalization and biological activity depends on specific sizes^1^,^4^,^15^,^16^,^17^. For instance, water soluble gold and silica nanoparticles have shown size-dependent cytotoxicity, some over narrow size ranges of less than a nanometer, even though bulk gold and silica are known to be biologically inert^1^,^16^. The major mechanisms by which nanoparticles are known to exert their toxicity on cells include (1) the generation of reactive oxygen species (ROS)^18^,^19^, (2) alteration of antioxidant enzyme activities, (3) disruption of mitochondrial function, (4) oxidative damage in DNA and subsequent disruption of DNA replication and transcription, and (5) disruption cellular membrane function, interfering with cellular integrity^4^,^20^,^21^,^22^,^23^,^24^,^25^,^26^,^27^.

In the case of lipophilic nanoparticles delivered by lipid vectors, there is evidence that particle size, composition and method of formulation influence the encapsulation of the nanoparticles into liposomes and micelles, thereby affecting delivery efficiency^28^,^29^,^30^. Lipophilic nanoparticles’ use in consumer products and potential applications by the therapeutics industry have created a demand for understanding of their interaction with the lipophilic regions of liposomes, micelles and living cells, for applications such as enhanced delivery of therapeutic agents^15^,^31^,^32^,^33^, while also providing a model for nanoparticle interaction with biological membranes^34^,^35^,^36^,^37^.

Traditional methods of encapsulation of hydrophobic nanoparticles include hydration of dry film mixtures of phospholipids and nanoparticles, or the use of buffered detergent dialysis of the nanoparticles into the already formed liposome. Both methods result in the nanoparticles either being embedded in the hydrophobic bilayer region of the liposome or being coated by a lipid bilayer to form a micelle^29^,^30^. Previous work has postulated and demonstrated a limit of about 6 nm on the size of the nanoparticles encapsulated, but recently larger aggregates of multiple nanoparticles about 60 nm in size have been incorporated into liposomes by pre-incorporation clustering of the nanoparticles^29^,^30^,^38^,^39^,^40^,^41^.

Semiconductor nanoparticles present promising uses for light-emitting applications^14^,^42^,^43^,^44^. These semiconductor nanoparticles are generally composed of a core/shell with the core usually from groups II-VI or III-V^2^. These heavy metals, however, can be toxic even at low concentrations when the cores are exposed to the intracellular environment^5^,^45^,^46^,^47^. Silicon represents a potentially non-toxic alternative due to the biocompatibility of bulk silicon, the optical properties of nanocrystalline silicon in the visible and near infrared spectrum, and the amenability of silicon’s porous form to loading with therapeutic agents^2^,^8^,^48^,^49^. In this study, we synthesize and size-separate allylbenzyl-capped silicon nanocrystals (ncSi:AB), as well as test the fraction-dependent encapsulation efficiency into liposomes and the accompanying cytotoxic effect they exert on HeLa cells.

## Results and Discussion

**Figure 1:**
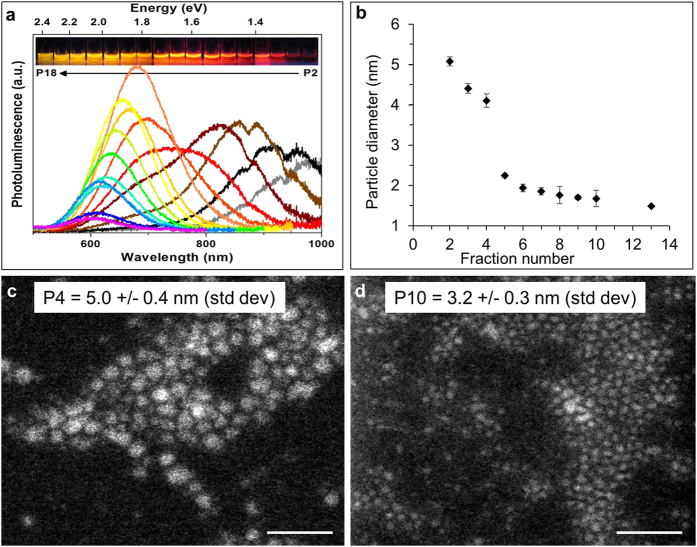
Separation and characterization of silicon nanoparticles. (**a**) Photoluminescence spectra of allylbenzyl-capped silicon nanoparticle (ncSi:AB) fractions show a blue shift in the photoluminescence maxima with increasing fraction number (and overall decreasing size); inset: photo of ncSi:AB fractions under UV illumination. (**b**) Plot shows size distribution of selected ncSi:AB fractions. (**c**) High-angle annular dark field scanning transmission electron microscopy (HAADF-STEM) image of fraction 4. (**d**) HAADF-STEM image of fraction 10. Scale bars = 20 nm.

In order to test the fraction-dependent toxicity of the ncSi:AB particles we first needed to size-separate the polydisperse mixture into monodisperse fractions and characterize them. Four batches of ncSi:AB fractions were used; batches A, B and C each containing 18 monodisperse and a polydisperse ensemble (ENS) fraction were used for the toxicity studies, while batch D, which contained 10 monodisperse and an ENS fraction, was used for the fluorescence co-localization studies. Figure 1a shows the photoluminescence (PL) characterization of fractions of size-separated ncSi:AB prepared by size-selective precipitation. Fraction A1, the first precipitated fraction, is not shown in Fig. 1a as the fraction displays cloudiness that prevents effective photoluminescence characterization. Successful size separation was confirmed by measuring the sizes of selected photoluminescent fractions using high-angle annular dark field scanning transmission electron microscopy (HAADF-STEM) as shown in Fig. 1b. Although not all fractions were characterized by HAADF-STEM, the selected fractions show the expected decreasing particle size with increasing fraction number (Supplementary Figure 1 shows size distribution of individually measured particle sizes). This trend, coupled with the blue shift with increasing fraction number (Supplementary Figure 2) which we demonstrated in previous work^2^, shows the reproducibility of the separation method. Representative images of fractions A4 and A10 are shown in Fig. 1c and d to indicate size difference between fractions. It is worth noting that in addition to size differences, two further explanations for the difference in PL observed include varying shape of the nanocrystal or a difference in surface coverage. Although we were unable to detect noticeable differences in shape or surface coverage from the HAADF-STEM data (see Supplementary Figure 3 as examples), we found this characterization sufficient for the first investigation of the effect of the size-separation procedure on the cytotoxicity of the nanoparticles.

The next step after separation was to encapsulate the nanoparticles in liposomes. To achieve this, we employed the buffered detergent dialysis method. Simply rehydrating a dried film of lipids and nanoparticles did not yield any detected encapsulation, as we could not re-solubilize the dried nanoparticle-lipid film during rehydration. Using a mixture of Hank’s Buffered Salt Solution (HBSS) with 1% β-octyl glucoside (OCG) to dissolve the dried nanoparticle film allowed for the re-solubilization of the nanoparticles before mixing with the already extruded liposomes. OCG functioned as a dialyzing agent used to solubilize the nanocrystals. This method resulted in larger nanoparticle-lipid complexes for the less toxic fraction A4 and smaller nanoparticle-lipid complexes for the more toxic fraction A10 (Supplementary Figure 4). The nanoparticle-liposome structures detected in our experiments showed a lipid monolayer coating of the clusters rather than incorporation into the lipophilic region of the already formed liposome. An interesting alternative to this approach which we did not pursue is single particle encapsulation into micelles rather than liposomes^50^,^51^.

**Figure 2:**
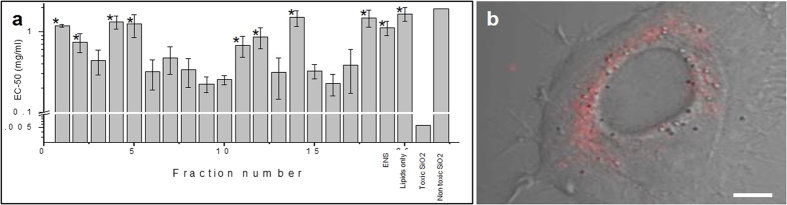
Low, reproducible toxicity of monodisperse ncSi:AB fractions. (**a**) Graph of EC50 plots showing toxicity of 18 fractions, the ENS, the lipid-only control and calculated EC-50 values of toxic and non-toxic SiO_2_ particles from the literature^15^. The EC-50 values for each fraction were compared between three batches using a student t-test to determine the reproducibility of EC-50 values. The error bars represent the standard deviation of the triplicate experiments for each batch of nanoparticles (A, B and C). Statistical comparison of the EC-50 values of the individual fractions using ANOVA and then the Tukey test on fractions A, B and C showed fractions 1, 2, 4, 5, 11, 12, 14, 18 and the ENS to be significantly less toxic (*) than the others (3, 6, 7, 8, 9, 10, 13, 15, 16, 17). (**b**) Merged fluorescence and bright field image of a single cell showing localization of nanoparticles in the cytoplasm around the nucleus at a lower concentration than the EC-50 value. Scale bar = 5 μm.

Cytotoxicity was measured for liposomal formulations of all 18 different ncSi:AB fractions and the ENS from batches A, B and C by determining the concentration that produces a half-maximal toxicity response (EC-50) on cultured HeLa cells (Fig. 2), with lower EC-50 values indicating higher cytotoxicity. Although most screening is currently done using a single dose^52^, we tested the nanoparticle toxicity over 3 dosages in order to account for any other effects beyond cytotoxicity that were concentration dependent. HeLa cells were found to be appropriate for this first step test due to their wide use in research as a standard initial model cell type. Supplementary Figure 5 shows toxicity measurements made on three different batches of size-separated ncSi:AB from batches A, B and C. Figure 2a is a combined graph of all three batches. We compared the measured EC-50 values for the monodisperse ncSi:AB nanoparticles with literature values obtained for toxic silica nanoparticles of about 14 nm from Napierska *et al*.^16^. The ncSi:AB particles showed a 100-fold lower toxicity than the toxic Stöber silica nanoparticles used in the Napierska study and an equivalent level when compared to the non-toxic Stöber silica particles of about 335 nm in diameter. The differences in toxicity between the smaller (<17 nm) silica particles used in the Napierska study and ours may arise from a difference in the ligands used and the possibility of the liposomes mitigating some of the potential toxic effects. The combined toxicities of the A, B and C ncSi:AB fractions all showed at least tenfold lower toxicity than 20 nm SiO_2._ These values are plotted along with our data to put the numbers into perspective, but it should be noted that the data from Napierska *et al*.^16^ were carried out on water soluble particles in a different cell line.

Although the overall cytotoxicity of ncSi:AB that we measured in HeLa cells is low, there were significant differences in the relative cytotoxicity of the different fractions. A one-way ANOVA was performed on the toxicity measurements and a significant difference was found between the fractions (p < 0.05). The one-way ANOVA only indicated a difference between the EC-50 values and hence a Tukey’s test was performed to compare each of the fractions with one another in order to determine which fractions specifically differed from one another. The EC-50’s of Fractions 1, 2, 4, 5, 11, 12, 14, 18 and the ensemble (ENS) from batches A, B and C were not found to be significantly different from one another or the lipid only control and were labeled the “less toxic”. Fractions 3, 6, 7, 8, 9, 10, 13, 15, 16, 17 from the same batches A, B, C were each found to be significantly different from the ones in the “less toxic group” and hence were labelled “more toxic”. This effect was reproducible between three repetitions of the experiment on each batch shown in Supplementary Figure 5. Interestingly, the differential cytotoxicity did not follow a monotonic trend with fraction number. Furthermore, the ensemble data showed a low toxicity comparable to the neat lipid controls, which suggests that specific cytotoxic nanoparticles may be enriched in the more toxic fractions.

Two possible explanations for the differential cytotoxicity are (1) specific particles may have specific effects within the cell^1^,^19^,^53^ and (2) specific particles may be taken up more efficiently by the cells^17^,^54^,^55^. To test these hypotheses, we first determined the cellular mechanism of toxicity to see if different fractions induced different effects within the cell. We then tested for oxidative stress and DNA damage in the cell. Although no DNA damage was detected (Supplementary Figure 6), both the less-toxic and more-toxic fractions showed a significantly higher level of oxidative stress than the negative control without ncSi:AB or lipids, the more toxic fraction causing higher oxidative stress (Supplementary Figure 6). There was no significant difference in fluorescence from the surface between the two tested fractions. The liposomes by themselves did not elicit an oxidative stress response significantly different from the negative control. These results indicate that all fractions resulted in some oxidative stress on the cell. The generation of reactive oxygen species by the ncSi particles, which could account for the oxidative stress, may occur via the Fenton-type reaction undergone by transition elements, which involves the generation of hydroxyl radicals from the interaction of exposed silicon surface sites with hydrogen peroxide in the cells^56^,^57^. Another possible mechanism is through the disruption of mitochondrial membranes resulting in endogenous production of reactive oxygen species^58^,^59^. The generation of free radicals has been reported to directly oxidize DNA, proteins and lipids, thus affecting cell function and integrity^6^,^7^,^60^. Since the mechanism of toxicity appeared to be similar for all particles, that is, they all induced oxidative stress, we next tested the hypothesis that the more-toxic fractions were taken up more than the less-toxic ones.

**Figure 3:**
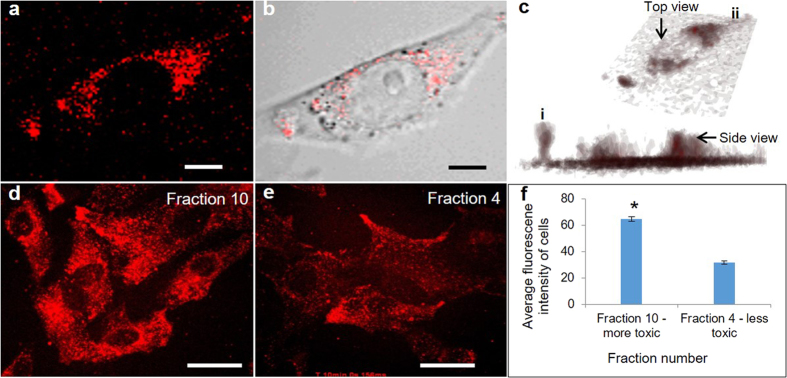
Cellular internalization of liposomally encapsulated ncSi:AB particles. (**a**) High magnification fluorescence image showing ncSi:AB particles delivered to a HeLa cell. (**b**) High magnification of merged fluorescence and bright-field images showing ncSi:AB particles delivered to a HeLa cell and localized around the nucleus of the cell. Scale bars = 5 μm. (**c**) Plot of z-stack fluorescence images taken of (**a**) showing internalization of nanoparticles and the exclusion of the nanoparticles from the nucleus. (**d**) High magnification confocal fluorescence image showing higher fluorescence intensity of ncSi:AB particles localized in HeLa cells for the more-toxic fraction A10. (**e**) High magnification confocal fluorescence image showing lower fluorescence intensity of ncSi:AB particles localized in HeLa cells for the less-toxic fraction A4. Scale bars = 20 μm. (**f**) Plot showing the difference in average fluorescence intensity of the internalized nanoparticles in 100 cells for each of fractions A4 (less toxic) and A10 (more toxic). *Indicates a significant difference between the two fractions using a student t-test, assuming the less-toxic fraction 4 as a control.

First, we selected fractions A4 and A10 as representative of the less toxic and more toxic fractions and examined their relative uptake by confocal laser scanning microscopy (Fig. 3). Figure 3a–c shows fluorescence images of ncSi:AB particles that have been taken up by a single cell, showing that the ncSi:AB particles are localized in the cytoplasm and are not present in the nucleus. Figure 3d and e are fluorescence images of the more-toxic fraction A10 and the less-toxic fraction A4, respectively. We quantified the amount of ncSi:AB internalized by the cells by measuring the average fluorescence intensities of 100 cells for each of fractions A4 and A10. The more-toxic fraction 10 showed significantly higher fluorescence intensity than the less-toxic fraction A4 (Fig. 3f). This suggests that the cells internalized the nanoparticles differently; the more-toxic fraction 10 internalized to a higher degree than the less-toxic fraction 4.

**Figure 4:**
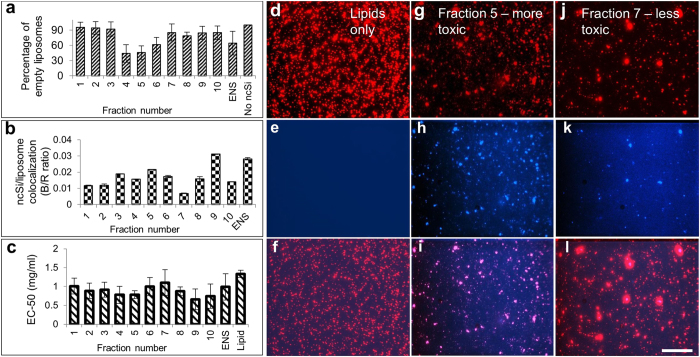
Fluorescence quantification of ncSi:AB brightness and liposomal encapsulation. (**a**) Plot of the percentage of empty liposomes as an indication of encapsulation efficiency of the fractions. The percentage of empty liposomes was obtained by finding the fraction of the liposomes observed in the TRITC channel that did not have any nanoparticles (UV channel) at the same location below a chosen threshold. (**b**) Ratio of blue (ncSi:AB) to red (TRITC-labeled liposome) normalized to the quantified (Supplementary Figure 4) brightness of the naked particles as a measure of encapsulation of the various fractions. (**c**) EC-50 toxicity measurements of the set of 11 monodisperse ncSi:AB from batch D used in the encapsulation study in A and B. (**d**–**f**) are the red (TRITC), blue (UV illumination and 580 nm long pass emission for ncSi:AB) and a merged image of both channels respectively for the lipid control with no ncSi:AB. G, H, I and J, K, L are the same channels as those used in D, E and F, but for fractions D5 and D7, respectively. Scale bar = 30 μm. Error bars represent standard deviations from triplicate samples. Comparison of the toxicity was done using a one-way ANOVA and a subsequent post hoc Tukey’s test as previously used in Fig. 2. Fractions D5 and D7 were chosen for comparison because they differed significantly from each other.

To further understand the reasons for the differential uptake, we tested the hypothesis that the more-toxic fractions were encapsulated more efficiently into the liposomes. We used fluorescence microscopy to quantify the encapsulation efficiencies of different fractions of ncSi:AB and compared the result to their cytotoxicity. For this part of the experiment a different batch of nanoparticles, batch D, was synthesized and separated. The separation resulted in 10 monodisperse fractions (Fig. 4) and an ENS fraction this time. Although this varies from the number fractions used in the initial toxicity study, it allowed us to confirm a fraction-dependent toxicity of the size-separated nanoparticles while also testing the encapsulation efficiency of the different fractions. Figure 4 shows fluorescence quantification of the co-localization of nanoparticles and liposomes. Figure 4a shows the percentage of empty liposomes as a measure of encapsulation efficiency. Figure 4b further charts the encapsulation of ncSi:AB in liposomes by measuring the co-localization of red (rhodamine-B doped liposomes) and blue (ncSi:AB) particles. Figure 4c shows the EC-50 measurement for the new set of fractions used for the fluorescence quantification. Figure 4d,g and j show rhodamine-labeled liposomes in the red channel for the lipid control, the more-toxic fraction 5 and the less-toxic fraction 7 from batch D. Fractions D5 and D7 were selected as they showed a significant difference in the measured EC-50 values (p < 0.05). Figure 4e,h and k show the absence of ncSi:AB particles in the lipid-only control, the presence of a high amount of ncSi:AB particles for the more toxic fraction D5 and fewer ncSi:AB particles for the less-toxic fraction D7. This suggests that more of the nanoparticles are encapsulated into the liposomes of the more-toxic fraction B5 than the less-toxic fraction D7, leading to the difference in toxicity to cells. Although it is unclear which fractions in D correspond to which fractions in A, B or C, the differences in the number of fractions obtained allowed us to test the reproducibility of our hypothesis of fraction-based enrichment of particles suitable for lipid encapsulation and subsequent cellular toxicity.

Supplementary Figure 4 shows the different types and sizes of liposome complexes formed by the less-toxic fraction 4 and the more-toxic fraction 10 from batch A. Fraction A4 produced larger (~80 nm diameter) silicon-impregnated liposomes with lower polydispersity measured using dynamic light scattering (Supplementary Figure 4) than fraction A10 which produced smaller liposomes (~67 nm diameter) with higher polydispersity (Supplementary Figure 4). Interestingly, neither average liposomal size (R^2^ = 0.0701) nor polydispersity (R^2^ = 0.2972) showed any correlation with fraction number, suggesting that nanoparticle interaction with liposomes and subsequent cellular uptake is unique to specific fractions and not predictable as a trend. Specifically, however, while the smaller liposomes of fraction A10 resulted in a larger quantity of liposomal nanoparticles delivered to the HeLa cells than did the larger liposomes formed by fraction A4, it is impossible to conclude that nanoparticle liposome size is the sole determinant of cellular internalization. This is because size-selective precipitation, like some other separation methods, may enrich for other properties like shape and ligand density, which could also affect nanoparticle interactions with biomolecules. This is especially true as Banerjee *et al*. have demonstrated that both nanoparticle uptake efficacy and transport are not only dependent on size and shape but also are cell-specific^61^. It is these new nanostructure-function relationships arising only after size separation, which make the separation and purification both essential and interesting. Our finding here of fraction-based differences in liposomal interaction of the silicon nanocrystals has implications in the liposomal delivery of nanomaterials, as well as in the understanding of size-dependent biological activity.

For instance, liposomes are often used as model cell membranes, and the ability of the more-toxic fractions to disperse better in liposomes suggests that they may also disperse better in biological membranes. Since disruption of mitochondrial membranes is one source which leads to oxidative stress, lipophilic nanoparticles may exhibit fraction-dependent effects within the cell due to the enrichment of particles with specific properties. Although the size-separated fractions are significantly more monodisperse than the ensemble, they too are a combination of particles of various sizes. Additional separation and characterization of the fractions may further enrich the more cytotoxic fractions and provide more insight into the mechanisms of nanomaterial interactions with biological systems. Further studies are therefore planned to fully characterize the nanoparticles in order to expand on and fully determine the rules that govern biological interactions of the monodisperse nanoparticles.

## Conclusion

Overall, the low toxicity shown by the silicon nanoparticles in this study represents a promising step forward in the drive to produce non-toxic nanomaterials. The increase in surface area of nanoparticles with decreasing size, which has accounted for the trend of higher toxicity of smaller sized nanoparticles^4^, does not seem to apply here. We further detected that within the range of nontoxicity of the silicon nanoparticles, there was a reproducible fraction dependence in the effects they had on the HeLa cells, which may depend on factors including but not limited to nanoparticle size. This fraction-dependent effect appears to be related to the specific interaction with liposomes that can affect their encapsulation efficiency and hence the efficiency of internalization should they be used in the hydrophobic form. This work demonstrates the value of further purification and screening of synthesized nanoparticles and opens the possibility of screening lipophilic nanomaterial drugs and libraries^62^,^63^,^64^.

## Materials and Methods

### Nanoparticle synthesis

The ncSi:AB were synthesized according to the previously reported sol-gel preparation with slight modifications^65^. Briefly, 10 mL of HSiCl_3_ (13.4 g, 99 mmol) was mixed with distilled water (40 mL, 2.22 mol) in a dry ice/acetone bath (−78 °C), forming a white solid (HSiO_1.5_)_n_ sol-gel glass. After washing with distilled water, filtering and drying it under vacuum, the solid (HSiO_1.5_)_n_ was thermally processed in a slightly reducing atmosphere (5% H_2_/95% Ar) to a peak processing temperature of 1100 °C at 18 °C/min and maintained there for 1 hour. The resulting light brown solid ncSi/SiO_2_ composite was mechanically ground in a mortar and pestle. Then 1.20 g of composite was stirred with 20 ml of ethanol and 40 ml of 49% HF (aq) in a polypropylene beaker for 2.5 h to etch the SiO_2_ matrix, liberate the ncSi, and gradually decrease particle size. The hydride-capped ncSi were extracted from the aqueous etching mixture using 35 ml of allylbenzene. Dissolved gases were removed by putting the flask under vacuum for about 20 min, then the solution was heated in an oil bath at 155 °C under a nitrogen atmosphere and left to stir for 17 hours. The resulting translucent orange solution was cooled and centrifuged for 20 min at 6461 rpm. The transparent orange supernatant was collected, and the precipitate was discarded. The allylbenzene was removed by vacuum distillation, and the solid was heated to 150 °C for 4 hours to remove any excess traces of the ligand. The polydisperse allylbenzyl-capped silicon nanocrsytal (ncSi:AB) solid was re-suspended in 10 ml of anhydrous toluene to give a clear orange solution.

### Nanoparticle separation and photoluminescence measurements

Size-selective precipitation was performed by repeatedly adding methanol to the ncSi:AB dispersion, followed by sonication for 20 s, centrifugation for 10 min at 6461 rpm, then decanting the clear solution from the solid precipitate. Solid ncSi:AB fractions were transferred directly into a glovebox and dispersed in 0.75 ml of toluene. This process was repeated with the supernatant until all fractions had been collected. UV-vis absorption spectra of the ensemble and fractions of ncSi:AB were obtained on a Perkin Elmer Lambda 900 UV/VIS/NIR spectrometer. Photoluminescence spectra of the ncSi:AB ensemble and fractions were obtained by exciting toluene solutions in an integrating sphere (Gigahertz Optik, custom made) with light from a 365 nm LED (Thorlabs M365L2), using a 1 mm diameter optical fibre (Ocean Optics) for collection and an Ocean Optics Maya 2000 spectrometer for detection. Three sets of mass-normalized samples were isolated by transferring specific amounts of each ncSi:AB dispersion based on the absorbance at 450 nm (average recorded between 449–451 nm) of each fraction to a new clean vial. All samples were left to dry for 3 days in a vacuum desiccator, then transferred to a glove box to be sealed under an inert atmosphere until used for the toxicity measurements.

### Electron microscopy

High-angle annular dark field scanning transmission electron microscopy (HAADF-STEM) was performed using an image-corrected FEI Titan 80–300 electron microscope. The microscope was operated at 300 kV, providing a nominal resolution of 0.14 nm. The ncSi:AB samples were drop-cast from toluene onto holey carbon-coated copper grids, which were further coated with 2 nm carbon film (Quantifoil). The HAADF-STEM images were processed by the non-linear anisotropic diffusion algorithm implemented in IMOD4 V4.1.4 for noise reduction prior to further analysis. Particle size measurement was done in imageJ by first calibrating each image using the scale from the original TEM image. A freehand ROI was drawn around a selected group of tightly packed particles to determine the overall area. This area was then divided by the number of particles within the ROI (Supplementary Figure 7). This process was repeated for multiple images for each fraction number and a standard deviation calculated. Particles were chosen for measurement based on experience to determine clarity of particle. For single particle distribution, each visible particle was measured by drawing lines which were roughly perpendicular to each other from one edge of a particle to the opposite edge. The average of the perpendicular diameters was then calculated and taken as the individual particle size (Supplementary Figure 7). A histogram was then made of the particle size distribution.

### Liposome formulation

1,2-dioleoyl-*sn*-glycero-3-phosphocholine (DOPC), 1,2-dioleoyl-*sn-*glycero-3-phospho-L-serine (sodium salt) (DOPS) and cholesterol in chloroform were mixed in a glass vial in the ratio 5.5:3.5:1. Liposomal formulations of the different fractions were made by first preparing the liposomes using the hydration method, sonicating the liposomes, and then extruding them through a 0.1 μm membrane filter. A selected nanoparticle fraction dissolved in chloroform was aliquoted into a class vial and dried in a vacuum overnight. The dried film was then re-solubilized by adding a mixture of HBSS (without calcium or magnesium) and β-octyl glucoside, sonicating at 60 °C for 10 minutes and filtering 4 times through a 100 K Amicon^®^ Ultra centrifugal filter purchased from Millipore^TM^. The extruded liposomes were then added to the nanoparticle fractions and the mixture sonicated. This allowed for the lipids to coat aggregates of nanoparticles, as shown in Supplementary Figure 4B for the non-toxic fraction 4 and Supplementary Figure 4C for the more-toxic fraction 10. This method, a slight variation on existing encapsulation methods^29^,^30^, was employed because standard encapsulation methods involving the mixing of the lipids and hydrophobic nanoparticles in organic solvent before drying and reconstitution in buffer failed to yield any delivery into the cell.

### Cell toxicity assay

Hela cells were purchased directly from the American Type Culture Collection. Cells were plated in a tissue culture plate 24 hours prior to the experiment so that they were at a confluence of 70% at the time of incubation with the liposomes. To determine uptake, the cells were imaged 2 hr after incubation with liposomal nanoparticles. For toxicity, the cells were incubated with the liposomal nanoparticles for 48 hours and then stained with DAPI for cell viability counting.

### Statistics

The dose response experiments for each fraction were done in triplicate. To determine whether there was a difference in the toxicity measurements of the different fractions, a one-way ANOVA was performed on all the fractions. If a p value of <0.05 was obtained, then a Tukey’s test was conducted to compare the individual EC-50 values to see which ones specifically differed from one another. The group of fractions with lower EC-50 values was labeled “more toxic” while the group with the higher EC-50 values was labelled “less toxic”. A fraction from each group was then chosen for use in the “more toxic” versus “less toxic” comparisons.

### Determination of mechanism of toxicity

In order to observe the nucleus for morphological changes, treated cells were stained with DAPI and compared to control cells with liposomes only and with media only. Cellular hypoxia and oxidative stress levels were determined using the hypoxia/oxidative stress kit from Sigma Aldrich.

### Differential Scanning Calorimetry (DSC) measurements

The DSC measurements were performed using the (TA) Nano-DSC from Thermal Analysis with the autosampler. The scan rate of 1 C°/min was set with a target temperature range from T = 10 C° to 70 °C. Baseline scans were acquired by scanning the buffer solvent. Ethanol, the detergent Contrad 50, and water were used in the cleaning process. The extra water scan was executed before and after each sample run to make sure that an identical thermogram was obtained. Sample concentration of 1 mg/mL at a volume of 1 mL was used for all measurements.

### Dynamic Light Scattering measurements

The scattered light intensity was measured using a Malvern Zetasizer Nano ZS (Malvern, Herrenberg, Germany) equipped with a 633-nm He-Ne laser and operating at an angle of 173°. The Dispersion Technology Software, from Malvern, was used to collect and analyze the data. The measurements were made at a controlled temperature of 25 °C, on 500 μl of each sample measured in single-use polystyrene cuvettes (Fisher Emergo, Landsmeer, The Netherlands) with a pathlength of 10 mm. The intensity size distribution, the Z-average diameter (Z-ave) and the polydispersity index (PdI) were obtained from the autocorrelation function. The default upper threshold of 0.01, lower threshold of 0.05, and the default filter factor of 50% were used.

## Additional Information

**How to cite this article:** Kusi-Appiah, A. E. *et al*. Enhanced cellular uptake of size-separated lipophilic silicon nanoparticles. *Sci. Rep.* **7**, 43731; doi: 10.1038/srep43731 (2017).

**Publisher's note:** Springer Nature remains neutral with regard to jurisdictional claims in published maps and institutional affiliations.

## Supplementary Material

Supplementary Information
