# Supplementary material for: Enhanced cellular uptake of size-separated lipophilic silicon nanoparticles
Source: Sci Rep. 2017 Mar 8;7:43731. doi: 10.1038/srep43731 (PMC5341124; doi:10.1038/srep43731)
Supplement: Supplementary Information [file srep43731-s1.pdf]

Enhanced cellular uptake of size-separated lipophilic silicon nanoparticles

Aubrey E. Kusi-Appiah[1], Melanie L. Mastronardi[2], Chenxi Qian[2], Ken Chen[2], Lida Ghazanfari[1], Plengchart Prommapan[3], Christian Kübel,[5] Geoffrey A. Ozin[2*], Steven Lenhert[1*]

Supplementary information

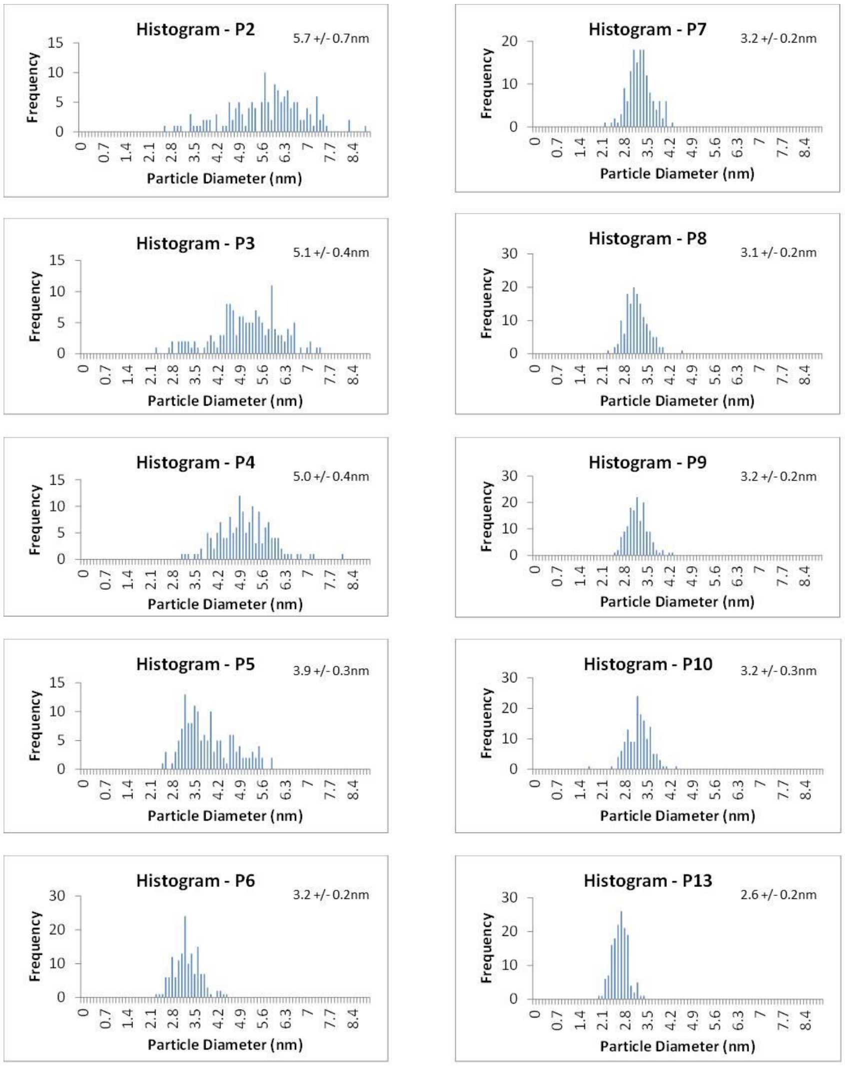

Supplementary Figure 1. Histograms outlining particle size measurements of the photoluminescent fractions imaged using high angle annular dark field scanning transmission electron microscopy (HAADF-STEM). Average diameter and standard deviation shown in top right corner of each histogram.

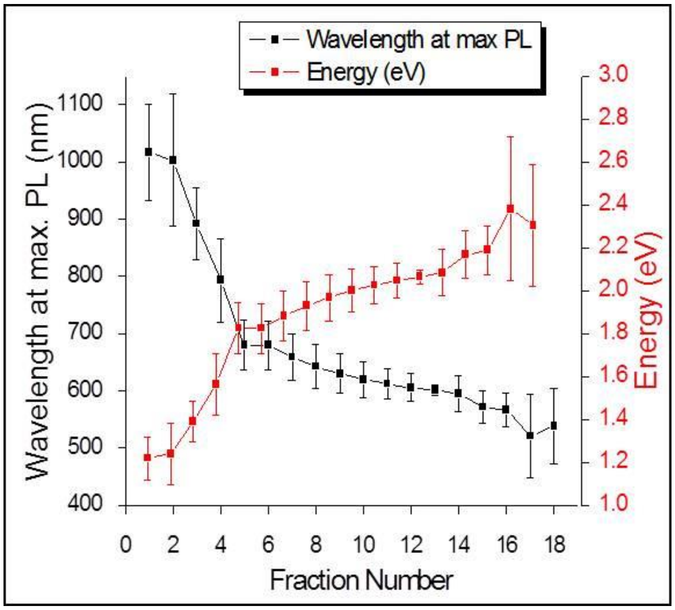

Supplementary Figure 2. Characterization of size separated ncSi particles. Plot shows reducing energy with decreasing fraction number (increasing particle size) shown in red. Black plot shows decreasing photoluminescence maxima wavelength with increasing fraction number (decreasing particle size). Maximum photoluminescence obtained from Gaussian fit of photoluminescence vs wavelength. Gaussian fit used was $Y = y0 + (\frac{A}{w\times\sqrt{\frac{PI}{2}}}) \times e^{(-2(\frac{x-xc}{w})^{2}}$. Energy values calculated from PL maxima. (C) Red shift of PL maxima with increasing particle size.

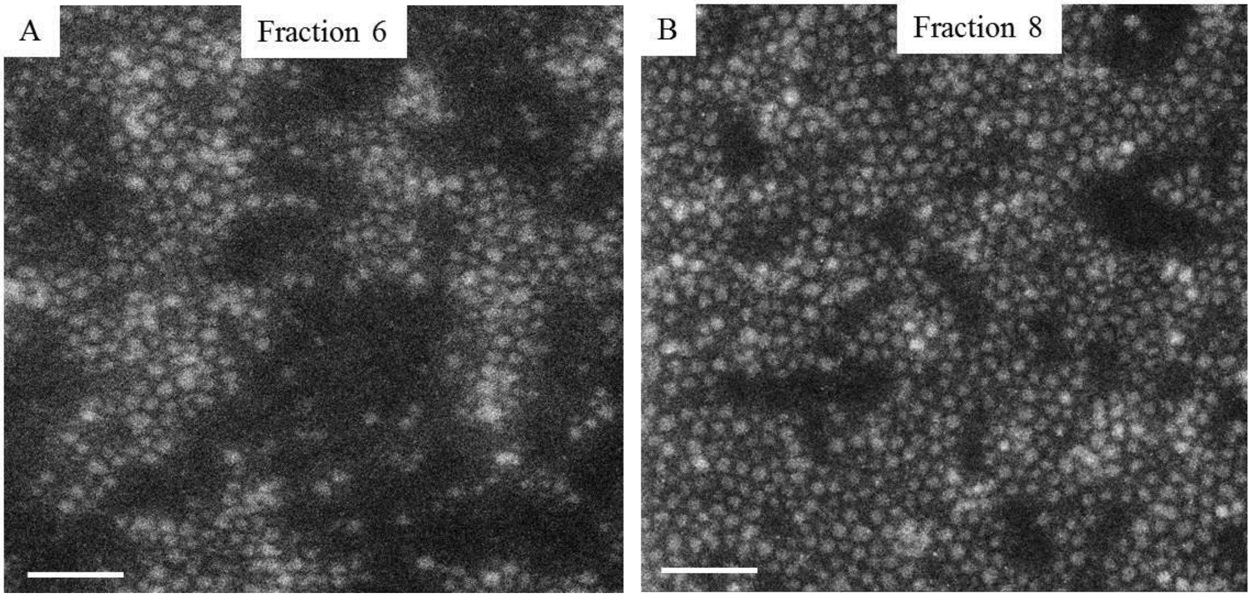

Supplementary Figure 3. High-angle annular dark field scanning transmission electron microscopy (HAADF-STEM) image of fractions A6 (a) and A8 (b) showing a non-cubic nanocrystal shape. Scale bar is 20 nm.

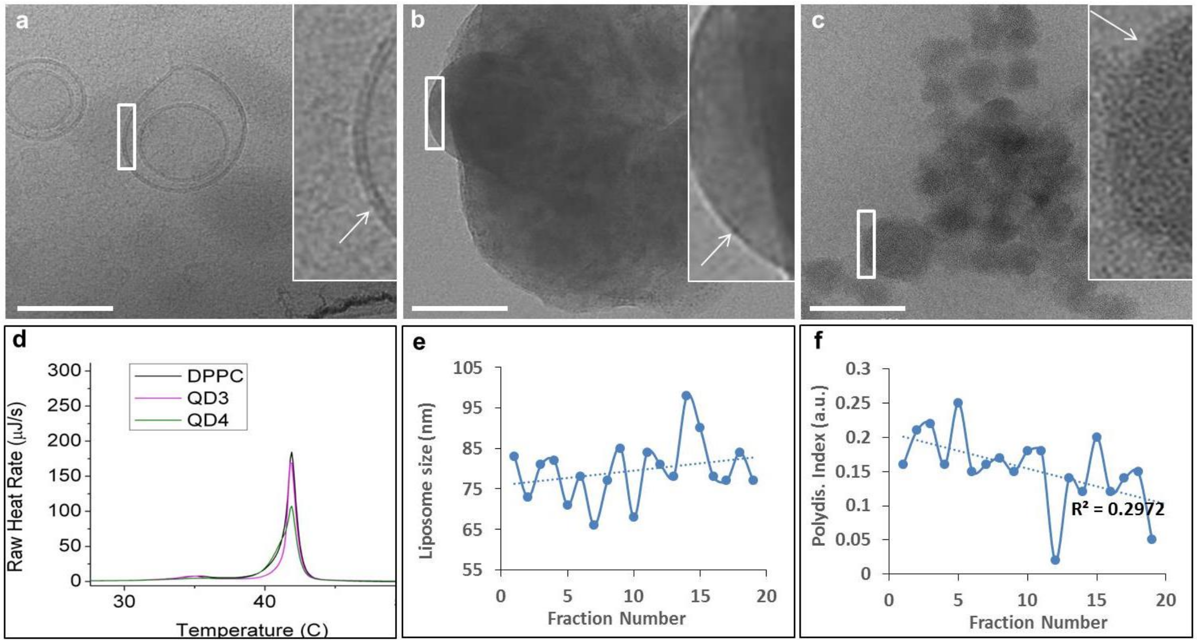

Supplementary figure 4. Characterization of liposomally encapsulated silicon nanoparticles (ncSi). (A) Cryo-TEM of empty liposomes. Scale bar – 100 nm. Inset is the indicated area zoomed in with arrows showing the lipid bilayers. (B) Cryo-TEM of lipids-ncSi fraction 4 complexes formed for use as a delivery vector. Inset is the indicated area zoomed in with arrows showing lipid monolayer. Scale bar is 50nm. (C) Cryo-TEM of lipids-ncSi fraction 10 complexes formed for use as a delivery vector. Inset is the indicated area zoomed in with arrows showing lipid monolayer. Scale bar = 50 nm. (D) Graph of the heat absorption of different liposomally encapsulated fractions of ncSi using differential scanning calorimetry (DSC). (E) Graph of size distribution of the liposomally encapsulated lipid ncSi fractions measured using dynamic light scattering (R^2=0.0701). (F) Graph of size-dependent polydispersity of the different fractions encapsulated with lipids (R^2=0.2972).

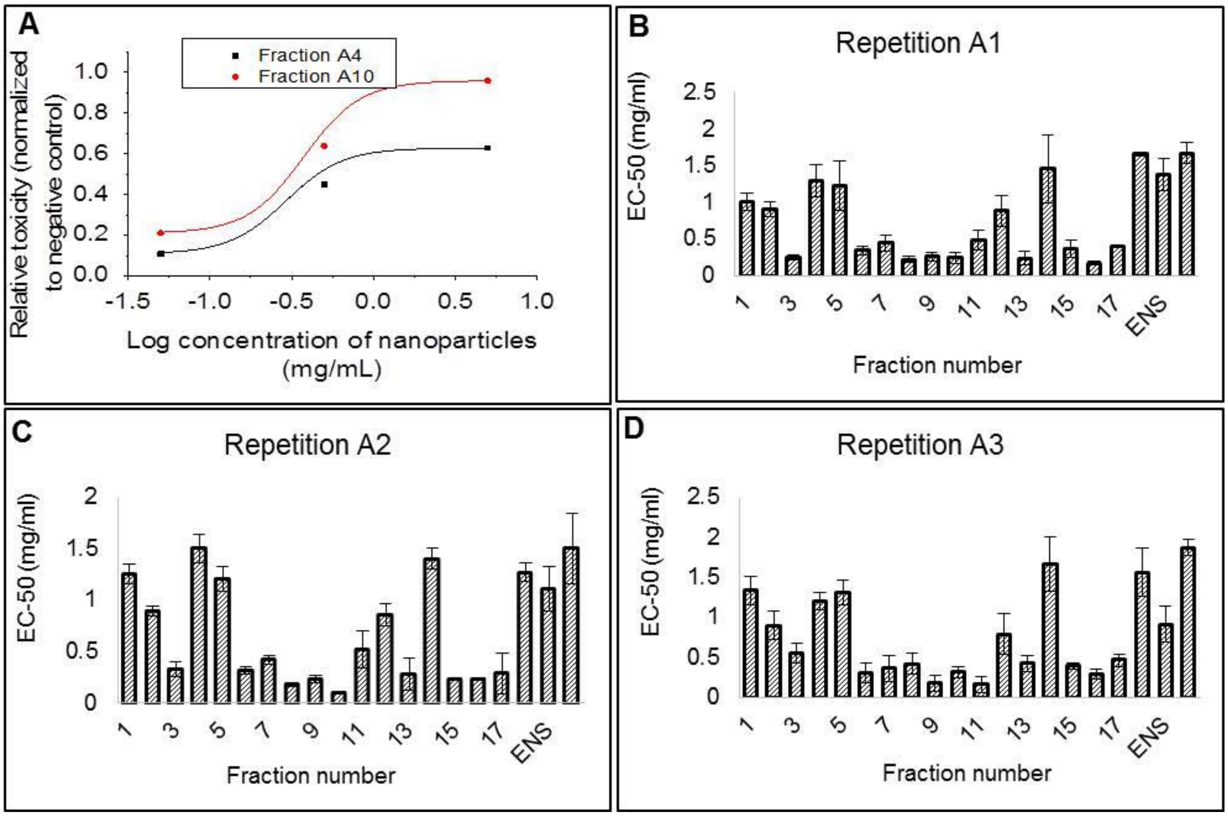

Supplementary figure 5. Reproducible EC-50 values from 3 different batches of nanoparticles. A shows representative dose response curves for the two factions, A4 and A10, chosen for comparison. (B), (C) and (D) are three different EC-50 toxicity plots from three different batches of synthesized and size-separated ncSi particles. Each experiment was done in triplicate. The error bars represent the standard deviation of the triplicate experiments for each batch of nanoparticles.

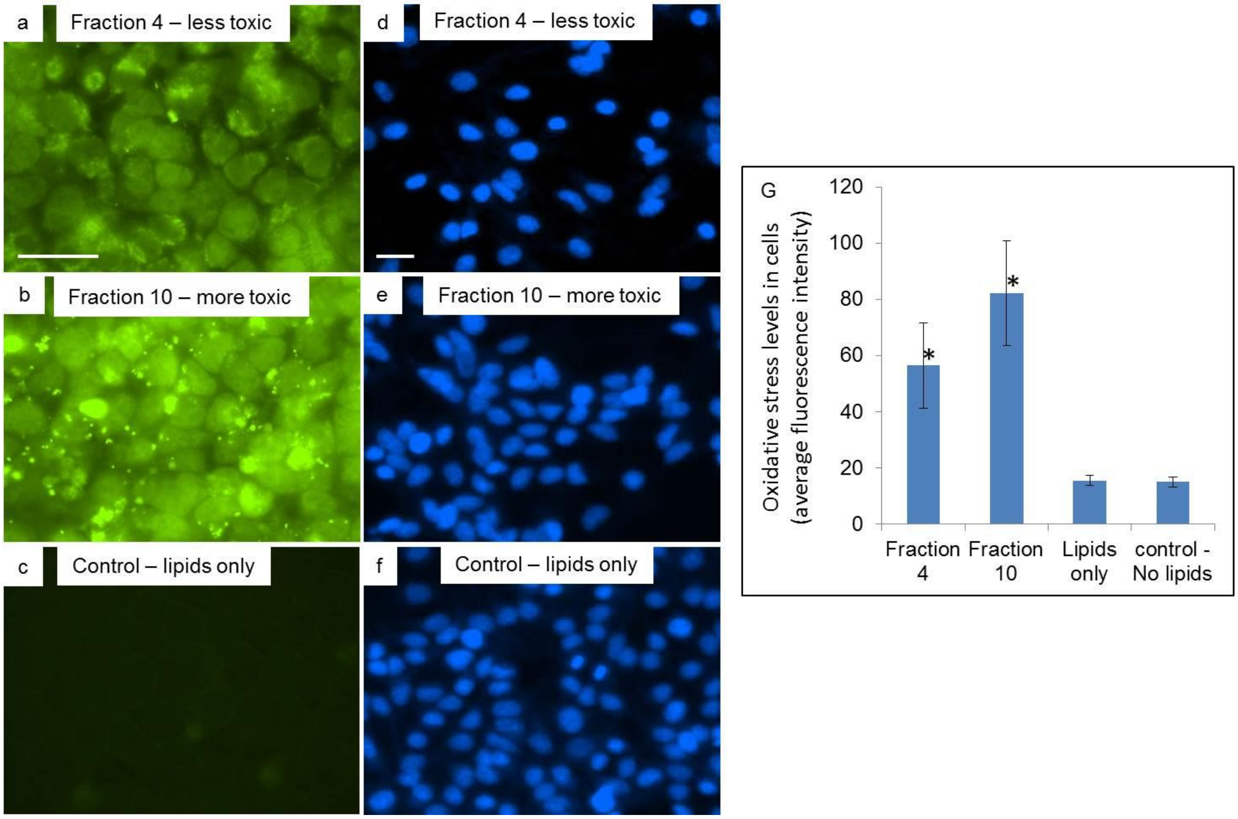

Supplementary Figure 6. Mechanism of toxicity of ncSi particles. (A) FITC fluorescence image showing level of oxidative stress in the cell caused by the less toxic fraction 4. (B) FITC fluorescence image showing level of oxidative stress in the cell caused by the more toxic fraction 4. (C) FITC fluorescence image showing level of oxidative stress in the cell caused by the lipids only. (D), (E), (F) are fluorescence images showing the nuclei cells incubated with fractions 4, 10 and lipids only respectively. No morphological differences found between nuclei. (G) Shows the oxidative stress undergone by the cells incubated with ncSi particles to be significantly greater (*) than those incubated with lipids only and those incubated with media only.

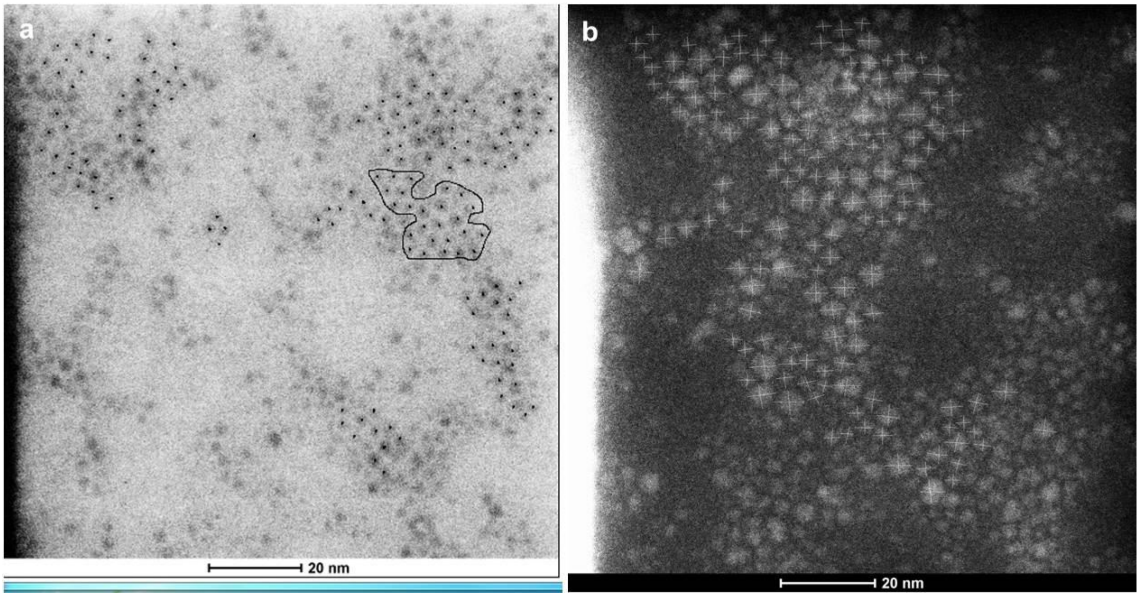

Supplementary Figure 7. Size analysis of size-separated ncSi nanocrystals. (A) Size measurement using freehand ROI method. (B) Size measurement by individual particle analysis
